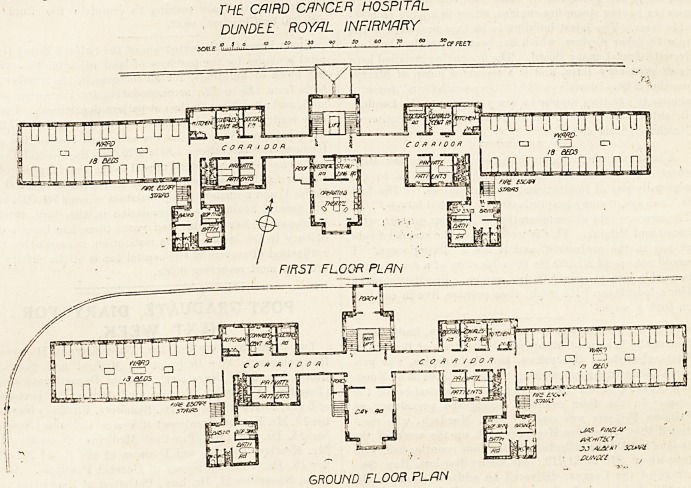# The Caird Cancer Hospital, Dundee Royal Infirmary

**Published:** 1907-11-09

**Authors:** 


### THE CAIRD CANCER HOSPITAL, DUNDEE ROYAL INFIRMARY.

THE building of which we give plans to-day owes its origin to the munificence of a Dundee manufacturer, Mr. J. K. Caird, LL.D. It is not only a hospital for the treatment of cancer, but a centre of investigation into the cause of the disease, and Mr. Caird's gift includes not only the building but the cost of the research department for five years. Although forming a part of the Dundee Royal Infirmary and under the same administration, it is a separate building, and may be regarded as a self-contained special hospital.

The plan has the merit of great simplicity; it consists of a central portion with two wings, the wings being connected to the central portion by covered bridges over the top of which the air has free circulation.

The ground at the disposal of the Committee not being of sufficient extent to permit of the wards being placed with their long axes north and south, the building had perforce to be planned so that one side of the wards face nearly due north. The aspect is not the best, but any other arrangement was clearly impracticable.

The centre part contains the staircase, in the well-hole of which is an electric lift provided with the automatic push-button arrangement. This now well-known form of lift is of special value in a hospital, as with it an attendant is not required. It has the further advantage of being very economical in working, the amount of current used per journey being very small. Security against accidents is provided for by the arrangement which makes it impossible to move the lift car up or down unless all the gates are shut and locked, while at the same time no gate can be opened unless the lift car is at rest immediately opposite it.

On the ground floor facing the staircase is a large day room, a most essential adjunct to a cancer hospital, where many of the patients are able to get up during the day. This room has a south aspect.

On the first and second floors the space over the day room is occupied by an operation theatre, with an anæsthetic room and a sterilising room adjoining.

The aspect of the theatres is not, of course, the best for the purpose, being, like that of the day room, nearly due south. But although a south aspect is not a desirable one for an operation theatre it is a possible one, while a north aspect for the day room would have been disastrous.

No attempt seems to have been made to get a top light for either of the theatres, and the arrangement of windows does not appear to have been very carefully thought out. The square bay window, while doubtless a great advantage to the day room, in the theatre can only have the effect of curtailing space just where it is most valuable.

The two ward wings are similar in all respects, and contain three floors of wards giving a total of 120 beds, twelve of which are in single rooms and intended for paying patients. The sanitary offices are well planned, and are effectually disconnected from the wards by cross-ventilated lobbies. The other rooms attached to the ward are a ward kitchen, linen room, room for the medical officer in charge, and a small pantry and store for patients' clothes. The floor area per bed is 106 feet and the cubic space 1,320 feet, the beds being spaced eight feet from centre to centre.

The whole building is warmed by means of low-pressure steam, there being no fireplaces. No special provision appears to be made for ventilation beyond the windows; the absence of open fireplaces is much to be regretted, both on the score of appearance and of the great value of radiant heat, and also of the extracting power of the smoke flue. The only thing to be said is that the system adopted is infinitely preferable to any scheme of forced draughts, "plenum" or otherwise. On the third floor accommodation is provided for the nursing staff.

Considerable care seems to have been taken with the whole of the details of the interior finishings, which would appear to have been largely modelled on the best modern work. A complete installation of electric apparatus and the equipment of both theatres, including the sterilising apparatus, forms part of Mr. Caird's noble gift. The cost appears to have been about £18,000, but no accurate information has reached us on this point. The building has been designed and carried out under the superintendence of Mr. James Findlay, architect.

## Figures and Tables

**Figure f1:**